# UBE2C cell-free RNA in urine can discriminate between bladder cancer and hematuria

**DOI:** 10.18632/oncotarget.11277

**Published:** 2016-08-12

**Authors:** Won Tae Kim, Pildu Jeong, Chunri Yan, Ye Hwan Kim, Il-Seok Lee, Ho-Won Kang, Yong-June Kim, Sang-Cheol Lee, Sang Jin Kim, Yong Tae Kim, Sung-Kwon Moon, Yung-Hyun Choi, Isaac Yi Kim, Seok Joong Yun, Wun-Jae Kim

**Affiliations:** ^1^ Department of Urology, Chungbuk National University College of Medicine, Cheongju, Chungbuk, South Korea; ^2^ Department of Urology, Myongji Hospital, Seonam University College of Medicine, Goyang, South Korea; ^3^ Department of Urology, Graduate School of Medicine, Hanyang University, Seoul, South Korea; ^4^ School of Food Science and Technology, Chung-Ang University, Anseong, South Korea; ^5^ Department of Biochemistry, Dongeui University College of Oriental Medicine, Busan, South Korea; ^6^ Section of Urological Oncology, The Cancer Institute of New Jersey, Robert Wood Johnson Medical School, New Brunswick, NJ, USA

**Keywords:** biomarkers, RNA, urinary bladder neoplasms, urine

## Abstract

**Background:**

There is growing interest in circulating nucleic acids as cancer detection biomarkers. Therefore, the aim of the present study was to identify a key urinary cell-free RNA marker that may assist in the diagnosis of BC.

**Results:**

Five cell-free RNAs were selected as candidate cell-free RNAs from tissue microarray data. An area under the curve (AUC) cut-off value of 0.7 in receiver operating characteristic (ROC) curve analysis identified four urinary cell-free RNAs for further analysis (CDC20, ESM1, UBE2C, and CA9; AUC = 0.716, 0.704, 0.721, and 0.702, respectively). Binary logistic regression analysis revealed that high expression of UBE2C was significantly associated with BC (OR, 1.754; CI, 1.147–2.682; *p* = 0.010). Analysis of UBE2C expression in urine samples from BC patients and hematuria controls yielded an AUC of 0.839, with a sensitivity of 82.5% and a specificity of 76.2%. UBE2C levels was significantly increased in G2 and G3 tumors compared to normal controls (*p* <0.001, respectively).

**Materials and Methods:**

Urine samples from 212 BC patients and 106 normal controls (64 healthy individuals and 42 with hematuria) were examined. The candidate cell-free RNAs identified from tissue microarrays derived from BC and normal control tissues was then measured in the urine samples.

**Conclusions:**

The levels of urinary UBE2C cell-free RNA were significantly higher in BC samples than in normal and hematuria control samples. The higher levels of urinary UBE2C cell-free RNA in BC might reflect high expression in BC tissues. Therefore, urinary UBE2C cell-free RNA may be a valuable diagnostic marker for BC.

## INTRODUCTION

Bladder cancer (BC) is the seventh most common cancer worldwide in men, and the 17th most common in women [1]. Every year, approximately 110,000 men and 70,000 women are diagnosed with BC, and about 17,000 patients in the United States and 40,000 patients in the European Union die from the disease [1].

Cystoscopy and urinary cytology are the gold standard diagnostic tools for BC. However, cystoscopy is both invasive and uncomfortable, and urine cytology has poor sensitivity [2, 3]. Therefore, a new noninvasive method with better sensitivity and specificity for detecting BC is necessary. Historically, a number of urine-based tests, including bladder tumor antigen (BTA), nuclear matrix protein 22 (NMP22), and fluorescence *in situ* hybridization (FISH), have been developed [4, 5]. Recently, new noninvasive diagnostic methods based on metabolomics and on measurement of microRNA markers in urine have been reported [6–8]. However, the diagnostic capability of BTA, NMP22, and FISH has been insufficient and further validation studies are necessary to confirm whether metabolomics and microRNA markers can be used detect BC.

Recent studies isolated circulating nucleic acids from body fluids such as serum, plasma, and urine [9–11]. Thus, there is a growing interest in circulating nucleic acids as cancer detection biomarkers. The origins of circulating nucleic acids remain unclear, but it is thought that they are released from apoptotic and necrotic cancer cells [12]. In bladder cancer, urine could be an ideal source of biomarker because it can experiences direct contact with cancer. In addition, the measurements of mRNA using RT-PCR in urine are reported to have a high sensitivity and specificity in BC patients [13].

The concentration of cell-free RNA in urine is three times higher than that of cell-free DNA [14]; therefore, the aim of the present study was to identify a key urinary cell-free RNA marker that may assist in the diagnosis of BC.

## RESULTS

### Baseline characteristics of the study population

The mean age of the 212 BC patients (177 males and 35 females) was 65.75 ± 12.69 years and that of the 106 controls (60 males and 46 females) was 64.46 ± 9.55 years. Forty-nine, 93, and 70 patients had grade G1, G2, or G3 cancer, respectively, and 65, 78, 31, 18, and 20 patients had stage Ta, T1, T2, T3, or T4 cancer, respectively. Other baseline characteristics are listed in Table 1.

**Table 1:** Baseline characteristics of the study patients

Variables (%)	UC cases	Controls	*p*-value	Healthy controls	Hematuria controls
Number	212	106		64	42
Mean age ± SD	65.75 ± 12.69	64.46 ± 9.55	0.359	65.77 ± 7.87	62.48 ± 11.48
Gender			< 0.001		
Male	177 (83.5)	60 (56.6)		35 (54.7)	25 (59.5)
Female	35 (16.5)	46 (43.4)		29 (45.3)	17 (40.5)
Grade					
G1	49 (23.1)				
G2	93 (43.9)				
G3	70 (33.0)				
T stage					
Ta	65 (30.7)				
T1	78 (36.8)				
T2	31 (14.6)				
T3	18 (8.5)				
T4	20 (9.4)				
N stage					
N0	189 (89.2)				
N(1–3)	23 (10.8)				
M stage					
M0	202 (95.3)				
M1	10 (4.7)				
NMIBC size					
< 3 cm	81 (56.6)				
≥ 3 cm	62 (43.4)				
NMIBC number					
Single	85 (59.4)				
Multiple	58 (40.6)				
MIBC prior radical cystectomy					
No	31 (44.9)				
Yes	38 (55.1)				

### Selection of candidate urinary cell-free RNAs from tissue mRNA array data

Table 2 lists the five candidate urinary cell-free RNAs selected from tissue mRNA array data derived from BC patients and normal controls. Eleven candidate genes were identified as the top ranked genes based on their increased levels in BC tissues compared with normal controls. However, six of these cell-free RNAs were not detected in urine by RT-PCR. Thus, five cell-free RNAs (CDC20, TOP2A, ESM1, UBE2C, and CA9) were selected as candidate BC detection markers.

**Table 2:** Candidate cell-free RNAs in urine^*^

Gene symbol	Tissue mRNA array (normal *vs*. BC)		Urine
	*p*-value	- fold change	Present or not
CDC20	*P* < 0.001	3.9323197	+^†^
TOP2A	*P* < 0.001	3.2166836	+^†^
ESM1	*P* < 0.001	3.1806504	+^†^
UBE2C	*P* < 0.001	2.9033677	+^†^
CA9	*P* < 0.001	2.6839344	+^†^
IQGAP3	*P* < 0.001	3.3673384	−
WDR72	*P* < 0.001	3.3154834	−
NUSAP1	*P* < 0.001	2.8828531	−
LOC440157	*P* < 0.001	2.8201986	−
TTK	*P* < 0.001	2.8054334	−
IGF2	*P* < 0.001	2.7653014	−

* These cell-free mRNAs were chosen because they showed the greatest increase of levels in bladder cancer tissue compared with controls on tissue microarrays.

† Presented candidate cell-free RNAs in urine.

### Levels of urinary cell-free RNAs in BC and normal control samples

**Figure 1:**
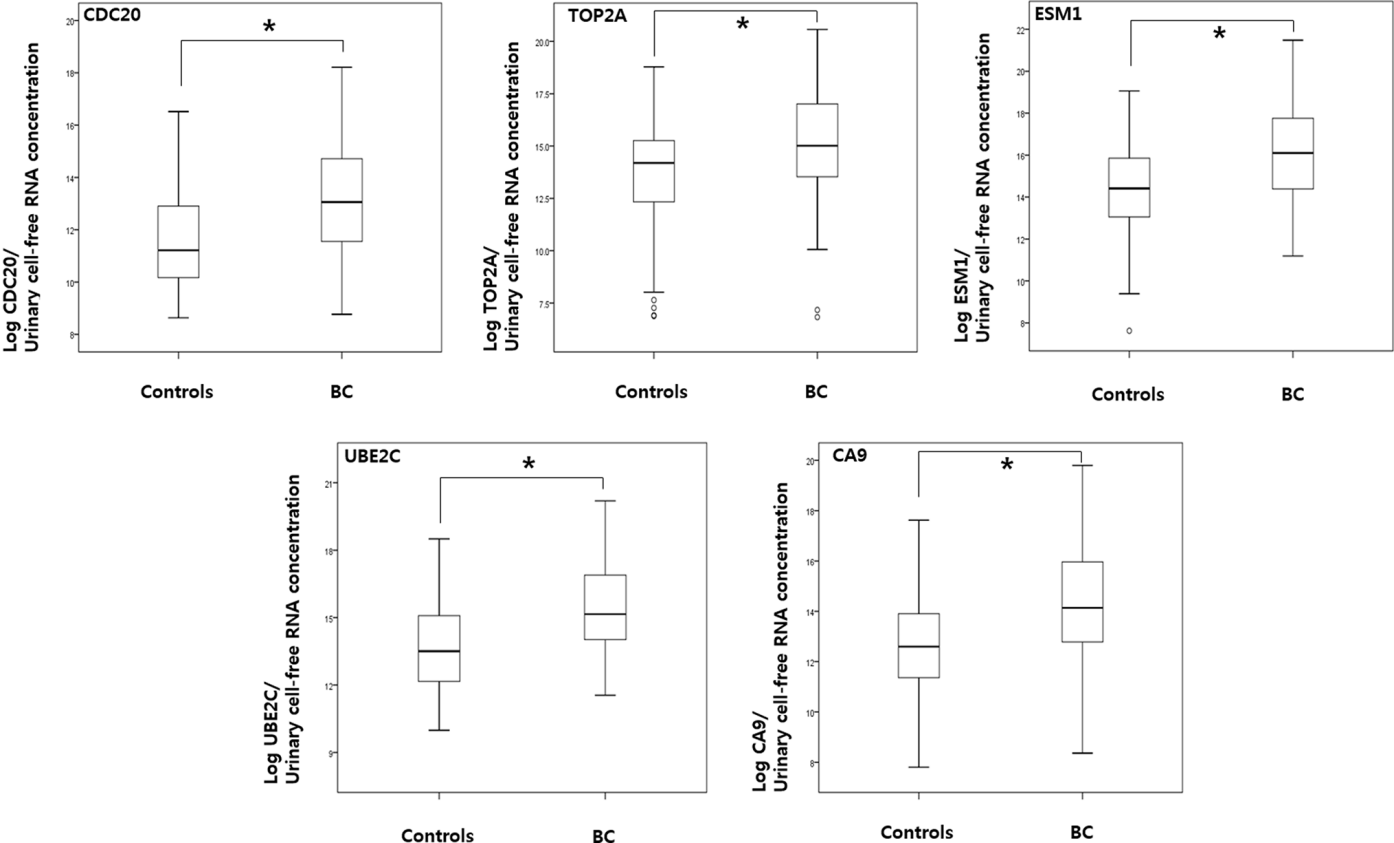
Comparison of urinary cell-free RNA expression levels in samples from bladder cancer patients and normal controls RT-PCR analysis of urinary cell-free RNA of CDC20, TOP2A, ESM1, UBE2C, and CA9 was performed as described in the Material and Methods section. *P* values were calculated using the Mann-Whitney *U* test. BC: bladder cancer.

As shown in Figure 1, the levels of CDC20, TOP2A, ESM1, UBE2C, and CA9 in urine samples from BC patients were significantly higher than those in samples from normal controls (*p* < 0.001, each). In addition, the levels of urinary cell-free RNAs from both NMIBC and MIBC patients were significantly higher than those from normal controls (*p* < 0.05, each) (Table 3).

**Table 3:** The levels of urinary cell-free RNAs between NMIBC or MIBC and normal controls

	Normal controls	NMIBC		MIBC	
Urinary cell-free RNAs	(the levels, × 10^4^copies/μg) Median (IQR)^a^	(the levels, × 10^4^copies/μg) Median (IQR)^a^	*p*-value^b^	(the levels, × 10^4^copies/μg) Median (IQR)^a^	*p*-value^b^
CDC20	9.6 (3.3–48.4)	27.7 (8.8–138.2)	< 0.001	156.5 (44.7–1144.5)	< 0.001
TOP2A	159.2 (24.5–454.1)	183.1 (44.1–1068.8)	0.022	1411.2 (302.5–8286.6)	< 0.001
ESM1	236.5 (49.3–916.5)	488.2 (138.8–2845.6)	< 0.001	4462.8 (827.5–18258.3)	< 0.001
UBE2C	95.6 (24.8–367.6)	208.8 (105.8–952.9)	< 0.001	1345.0 (368.7–9077.9)	< 0.001
CA9	34.1 (10.9–40.4)	78.9 (26.9–353.0)	< 0.001	705.7 (138.0–3918.6)	< 0.001

IQR interquartile range.

a Copy number of corresponding transcript per μg of total RNA used for cDNA synthesis.

b Mann-Whitney *U* test was used to compare expression between 2 groups.

### Selection of candidate urinary cell-free RNAs by ROC analysis

**Figure 2:**
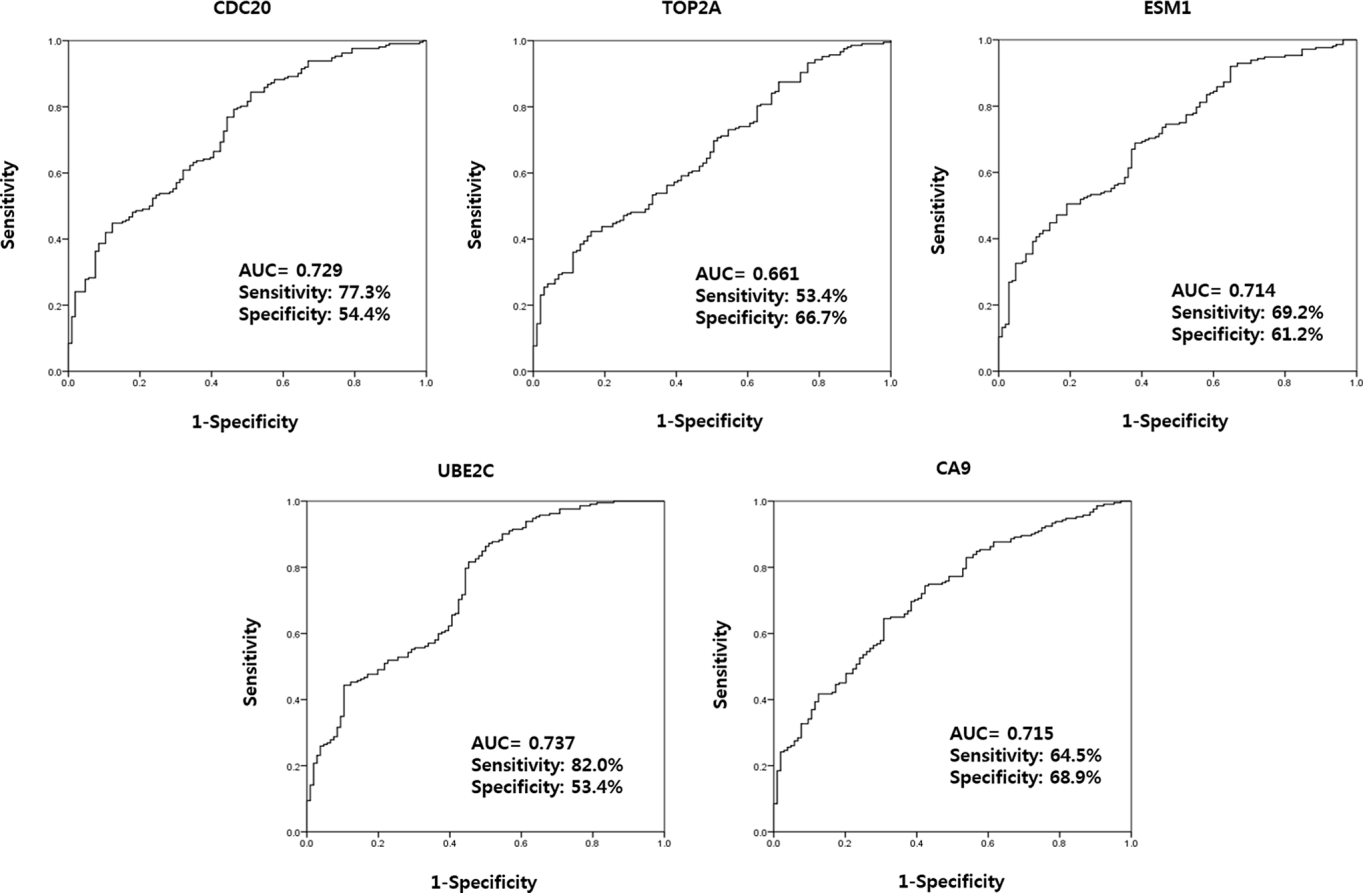
Receiver operating characteristic curve analysis of candidate urinary cell-free RNAs selected from microarray data The AUCs for CDC20, ESM1, UBE2C, and CA9 were 0.716, 0.704, 0.721, and 0.702, respectively. AUC: the area under curve.

ROC curve analysis led to the selection of four urinary cell-free RNAs (CDC20, ESM1, UBE2C, and CA9) that yielded an area under the curve (AUC) above the selected cut-off value of 0.7 (the AUCs for CDC20, ESM1, UBE2C, and CA9 were 0.716, 0.704, 0.721, and 0.702; respectively) (Figure 2). In addition, cut-off values for CDC20, ESM1, UBE2C, and CA9 (11.5101, 14.9784, 13.7558, and 13.4314, respectively) were selected based on the highest combined sensitivity (77.3%, 69.2%, 82.0%, and 64.5%, respectively) and specificity (54.4%, 61.2%, 53.4%, and 68.9%, respectively) for BC.

### Multivariate binary logistic regression analysis to select urinary cell-free RNAs as markers of BC

Multivariate binary logistic regression analysis of cell-free RNA levels in urine samples revealed that high UBE2C was significantly associated BC [odds ratio (OR), 1.754; confidence interval (CI), 1.147–2.682; *p* = 0.010] (Table 4). Based on the cut-off value for UBE2C (13.7558) obtained from ROC curve analysis, the level of UBE2C in urine samples remained significantly associated with BC (OR, 3.232; CI, 1.316–7.940; *p* = 0.011). These data suggest that urinary levels of UBE2C can be used to discrimination BC patients from normal controls.

**Table 4:** Multivariate binary logistic regression analysis to select the urinary cell-free RNAs as biomarkers for bladder cancer

Urinary cell-free RNA	Bladder cancer present			
	Logistic regression (according to expression value)		Logistic regression (according to cutoff value)	
	OR (95% CI)	*p*-value	OR (95% CI)	*p*-value
CDC20	1.013 (0.687–1.494)	0.947	0.999 (0.377–2.651)	0.999
ESM1	0.954 (0.693–1.314)	0.773	1.076 (0.458–2.525)	0.867
UBE2C	1.754 (1.147–2.682)	0.010^*^	3.232 (1.316–7.940)	0.011^*^
CA9	0.974 (0.735–1.292)	0.857	1.849 (0.836–4.086)	0.129

OR: Odds ratio, CI: confidence interval.

* The results of multivariate binary logistic regression analysis were confirmed by backward elimination.

### UBE2C can discriminate between BC and hematuria

**Figure 3:**
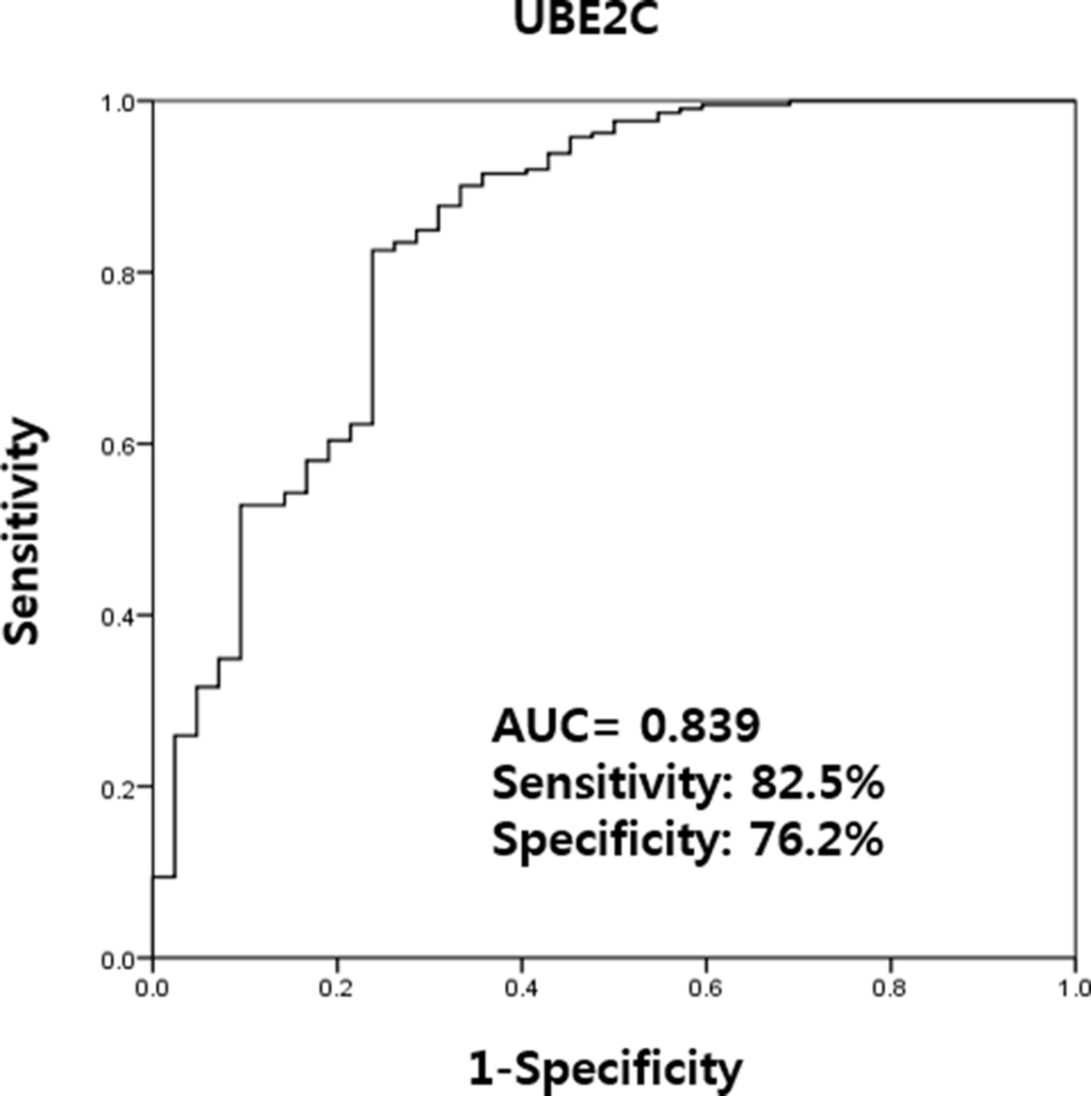
Receiver operating characteristics curve analysis of UBE2C urinary cell-free RNA levels in bladder cancer patients and those with hematuria from benign conditions Between BC patients and hematuria controls, the AUC of UBE2C was 0.839 and the sensitivity and specificity was 82.5% and 76.2%, respectively. AUC: the area under curve.

Urinary levels of UBE2C could be used successfully to discriminate BC patients from non-cancer patients with hematuria (AUC, 0.839; sensitivity, 82.5%; and specificity, 76.2%) (Figure 3).

### Urinary cell-free RNA, UBE2C as a valuable diagnostic marker in NMIBC and MIBC

**Figure 4:**
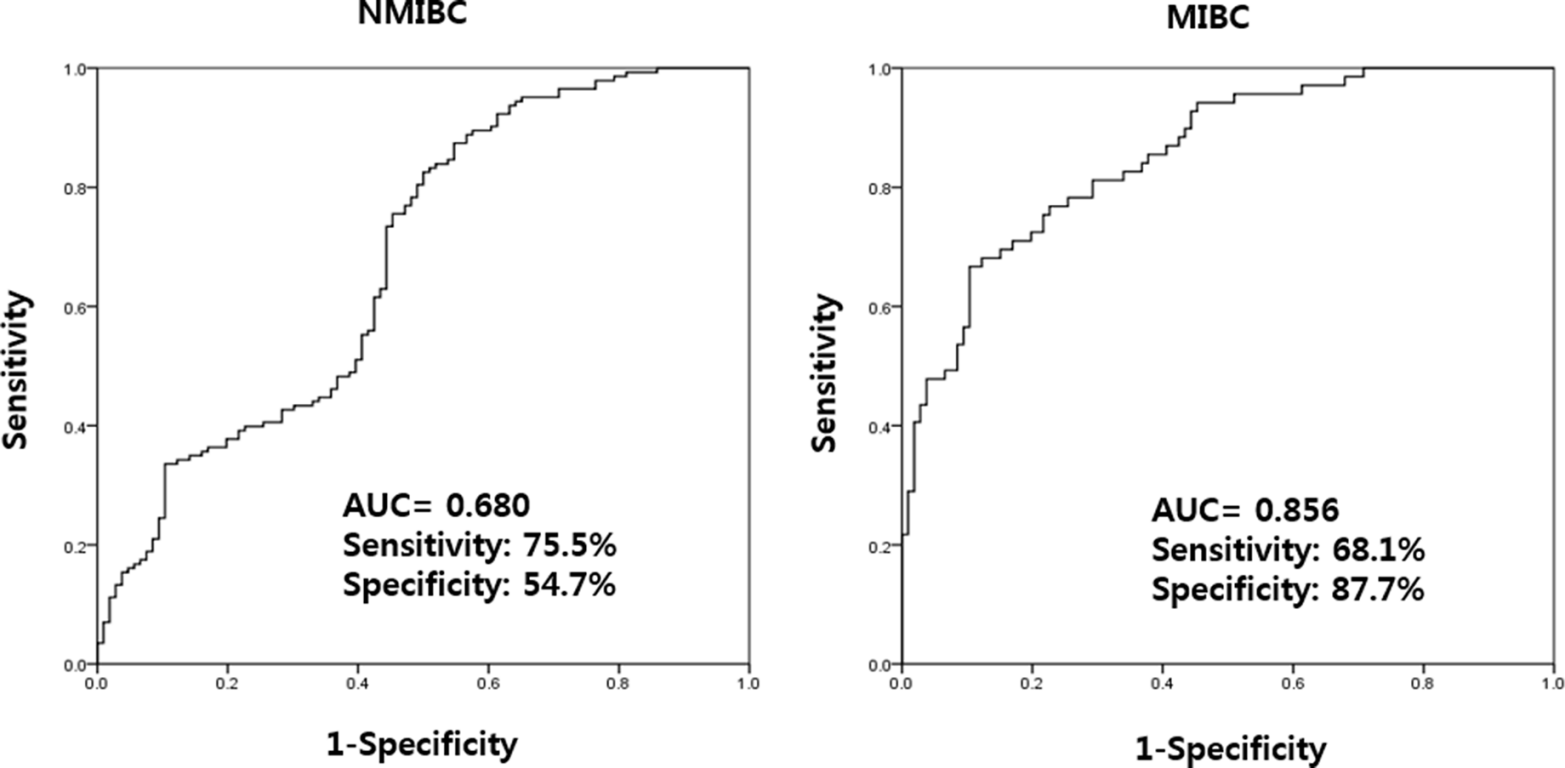
Receiver operating characteristics curve analysis of UBE2C urinary cell-free RNAs in NMIBC and MIBC The AUC values of NMIBC and MIBC were 0.680 and 0.856, respectively. AUC: the area under curve, NMIBC: non-muscle invasive bladder cancer, MIBC: muscle invasive bladder cancer.

In NMIBC and MIBC, higher UBE2C levels was significantly associated with NMIBC (OR: 1.501, CI: 1.279–1.763, *p* < 0.001) and MIBC (OR: 2.134, CI: 1.696–2.685, *p* < 0.001) (Table 5). Using a ROC curve, the AUC of UBE2C urinary cell-free RNAs in NMIBC and MIBC were 0.680 and 0.856, respectively (Figure 4). The highest combined sensitivity (specificity) of urinary cell-free RNAs of UBE2C in NMIBC and MIBC were 75.5% (54.7%) and 68.1% (87.7%), respectively.

**Table 5:** Binary logistic regression analysis of UBE2C cell-free RNA as a biomarker in NMIBC and MIBC

Urinary cell-free RNA	NMIBC		MIBC	
	OR (95% CI)	*p*-value	OR (95% CI)	*p*-value
UBE2C	1.501 (1.279–1.763)	< 0.001	2.134 (1.696–2.685)	< 0.001

OR: Odds ratio, CI: confidence interval.

### Urinary UBE2C levels according to tumor grade

**Figure 5:**
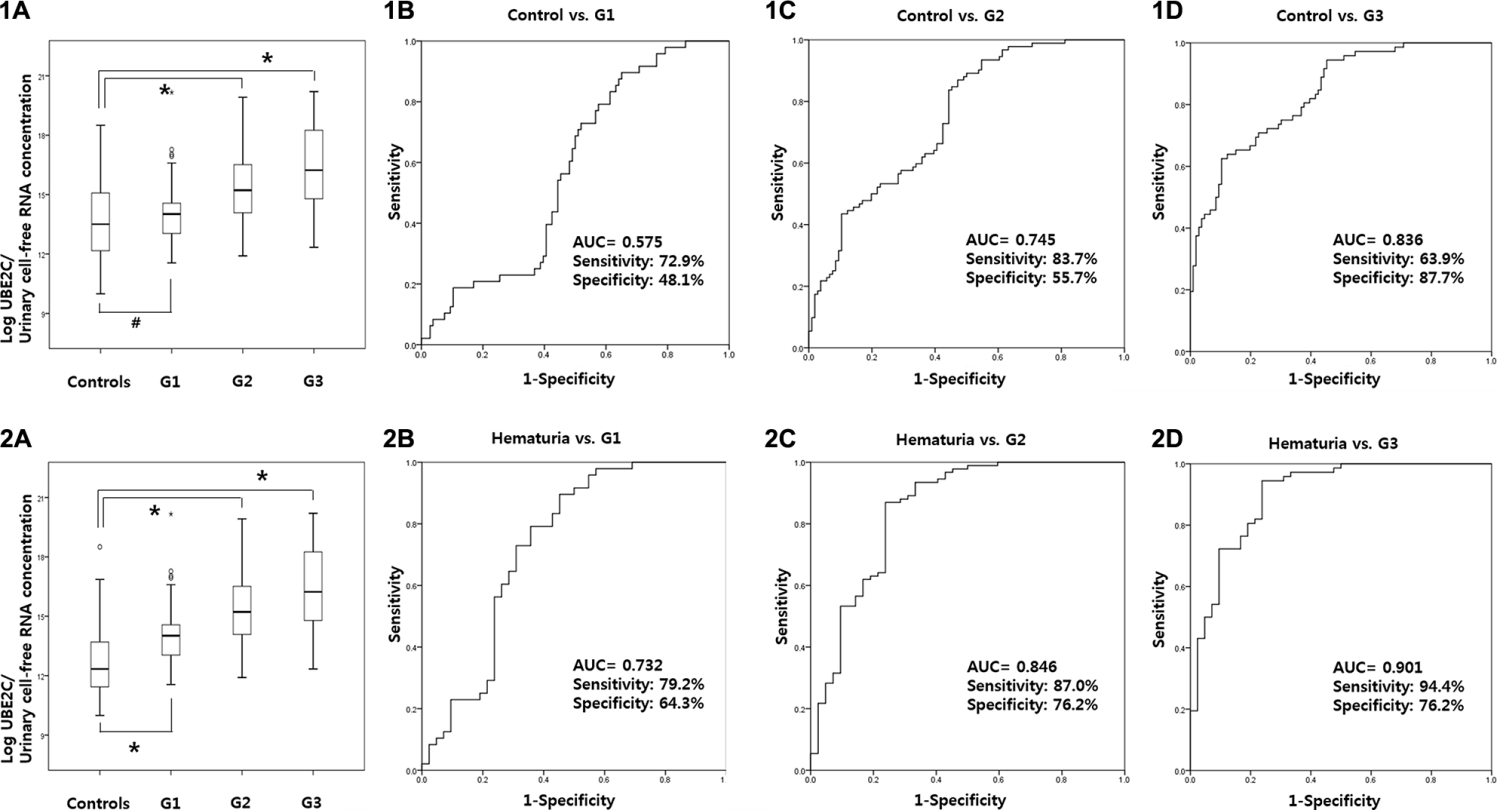
Urinary UBE2C levels according to tumor grade UBE2C levels was significantly increased in G2 and G3 tumors compared to normal controls (**1A**–**1D**). The AUC of UBE2C urinary cell-free RNAs according to tumor grade for G1, G2, and G3 were 0.575, 0.745, and 0.836 compared to normal controls. In comparison to hematuria patients, the AUC of UBE2C urinary cell-free RNAs according to grade G1, G2, and G3 were 0.732, 0.846, and 0.901 compared to hematuria patients (**2A**–**2D**). AUC: the area under curve.

UBE2C levels was significantly increased in G2 and G3 tumors compared to normal controls (*p* < 0.001, respectively). Using a ROC curve, the AUC of UBE2C urinary cell-free RNAs according to tumor grade G1, G2, and G3 were 0.575, 0.745, and 0.836, respectively, when compared to normal controls (Figure 5-1). In addition, the AUC of UBE2C urinary cell-free RNAs for G1, G2, and G3 were 0.732, 0.846, and 0.901, respectively, when compared to hematuria patients (*p* < 0.001, respectively) (Figure 5-2).

## DISCUSSION

The present study identified urinary levels of UBE2C cell-free RNA as a possible diagnostic marker for BC. Furthermore, measurement of urinary UBE2C levels is a suitable noninvasive tool for discriminating BC patients from non-cancer patients with hematuria. Thus, the present study is the first study to identify urinary UBE2C cell-free RNA as a diagnostic marker for BC.

UBE2C is a E2 ubiquitin-conjugating enzyme that promotes tumor cell growth and malignant transformation [15]. Okamoto et al. [15] reported that the expression of UBE2C is high in cancer cell lines but extremely low in normal tissues. Also, UBE2C expression is high in lung, stomach, and uterine cancer compared with surrounding normal tissues. Other reports show that expression of UBE2C is elevated in breast, thyroid, and colon cancer, and in lymphoma [16–18]. In addition, high expression of UBE2C correlates with cancer progression or a poor prognosis [19, 20]. In BC, high UBE2C expression is associated with a poor prognosis after radical cystectomy, and with higher tumor stage, lymphovascular invasion, and shorter cancer-specific survival [21]. Fristrup et al. [22] reported that UBE2C was an independent prognostic marker for progression from NMIBC to MIBC; these data were validated in Danish, Swedish, Spanish, and Taiwanese cohorts.

With respect to BC, urine is an ideal source of biomarkers because the bladder is a temporary urine reservoir; therefore, analysis of urinary nucleic acids may yield potential candidate diagnostic biomarkers for BC. Indeed, some studies show that urinary cell-free nucleic acids derived from urogenital cancer did not come from the blood; rather, they arose from tumor cells or their breakdown products [23, 24]. Recently, Casadio et al. [10] isolated urine cell-free DNAs (sequences [> 250 bp] derived from c-Myc, BCAS1, and HER2) from 51 BC patients and reported an AUC of 0.834 upon ROC curve analysis. Thus, they concluded that urine cell-free DNA is a potential marker for early noninvasive BC. Because this detection method is easy, noninvasive, and cheap, we suggest that it might open up new avenues for the application of urinary nucleic acids as biomarkers for BC.

Instead of urinary cell-free DNAs, urinary cell-free RNAs became the focus of our study. The source of urinary cell-free RNAs is the blood containing mRNA, rRNA, ncRNA, and miRNA, which can circulate as part of exosomes. These RNAs from the blood transport into urine through glomerular filtration, especially in bladder cancer. Subsequently, these RNAs in urine come in direct contact with the tumor. Bryzqunova et al. [14] reported that the concentration of urinary cell-free RNA was high, despite the fact that RNase activity was also high. It may be that urinary RNA is protected from nuclease-mediated degradation through forming complexes with biopolymers, as occurs in serum and plasma [25].

Here, we found that the levels of urinary UBE2C cell-free RNA were higher in BC patients than in normal controls (this result was consistent with that obtained from analysis of BC and normal control tissue arrays). Furthermore, urinary UBE2C cell-free RNA levels were higher in BC patients than in patients with hematuria (AUC = 0.839). This study follows our previous metabolomics study, which compared urine samples from BC patients and hematuria controls [7]. Unfortunately, however, no relationship between recurrence or progression and urinary UBE2C levels in NMIBC was observed (data not shown). With this, it is possible that the role of urinary UBE2C levels is limited to its diagnostic ability and that tissue analysis is a better prognostic marker in BC. Further studies on tissue marker for predicting prognosis in NMIBC are necessary.

The volume of urine produced by BC patient's fluctuates markedly depending upon the time of collection, hydration status, diet, and presence of renal disease. For example, examination of intraday differences in urinary RNA levels revealed a significant difference between morning and midday urine production [26]. Accordingly, the present study used RiboGreen as a reference gene to quantify total cell-free RNA levels in urine. In theory, RiboGreen-based quantification of total cell-free RNA in urine should make it easier to normalize the expression of each urinary cell-free RNA against that of a housekeeping gene; however, when we tried to normalize the expression of cell-free RNA against that of GAPDH, we found that the expression pattern and level of GAPDH was inconsistent. Thus, GAPDH is not a suitable reference for urine-based studies. To our knowledge, there is no previous study in BC that has conducted a direct measurements of urinary cell-free RNA with RiboGreen-based quantification.

The present study is also limited by a few weakness. This study is a single institution study with a limited number of participating patients. To address this, collaborations with other academic centers are being pursued and it is our hope that a large, prospective, randomized clinical trial on this topic will further validate our finding.

In conclusion, the levels of urinary UBE2C cell-free RNA were significantly higher in samples from BC patients than in samples from normal healthy individuals or from patients with hematuria. The higher levels of urinary UBE2C cell-free RNA in BC might reflects high expression of UBE2C mRNA in BC tissues. Therefore, urinary UBE2C cell-free RNA may be a valuable diagnostic marker for BC, although further confirmatory studies are required.

## MATERIALS AND METHODS

### Ethics statement

Collection and analysis of samples were approved by the Institutional Review Board of Chungbuk National University and each subject provided written informed consent (IRB approval number: 2010-12-010).

### Patients and urine samples

A total of 318 patients (212 BC and 106 normal controls) were enrolled in the study between May 2001 and September 2012. Age-adjusted controls comprised 64 healthy controls who visited the hospital for medical check-ups and 42 patients with microscopic hematuria due to non-malignant conditions (25 BPH patients and 17 SUI patients). Urine samples were collected in the morning and centrifuged at 25,000 rpm for 15 min. The supernatant and sediment were then placed into separate Eppendorf tubes and stored at −20°C until required. All tumor samples were primary tumors and these were obtained from patients who underwent transurethral resection (TUR) or radical cystectomy. All were histologically verified as urothelial transitional cell carcinoma. Tumors were staged and graded according to the 2002 American Joint Committee on Cancer TNM classification system and the 1973 WHO grading system, respectively.

### Extraction of cell-free RNA from urine

Urinary cell-free RNAs were extracted using the RNeasy Mini Kit (Qiagen GmbH, Hilden, Germany) from the supernatant urine after urine sediments had been removed by prior centrifuge. Each frozen urine sample (1 ml) was thawed at room temperature and treated with 1 ml of RLT buffer. One volume of 70% ethanol was then added to the lysate and mixed well by pipetting. The sample was then transferred to a column containing a membrane that binds cell-free RNA. The column was placed into a 2 ml collection tube and centrifuged for 15 s at 8,000 × g. The aqueous flow-through was discarded and the column replaced into the same collection tube. After addition of 700 μl of RW1 buffer, the column was centrifuged for 15 s at 8,000 × g and bound cell-free RNA was washed by addition of 500 μl of RPE buffer followed by centrifugation for 15 s. The aqueous flow-through was discarded and the column centrifuged for an additional for 15 s at 8,000 × g, after which it was placed into a clean 1.5 ml microcentrifuge tube. Cell-free RNAs were eluted by addition of 50 μl EB buffer followed by centrifugation for 1 min at 13,000 rpm. The cell-free RNAs were dissolved in EB buffer were stored at −20°C until required. cDNA was prepared from 1 μg of random primer-RNA using a RNA to cDNA EdoDry Kit (TAKARA BIO INC., Otsu, Japan), according to the manufacturer's protocol.

### Real-time PCR

Urinary cell-free RNAs were quantified by real-time PCR using a Rotor Gene 6000 instrument (Corbett Research, Mortlake, Australia). Real-time PCR assays were performed in micro-reaction tubes (Corbett Research, Mortlake, Australia) containing SYBR Premix EX Taq (TAKARA BIO INC., Otsu, Japan). The sequences of the gene-specific primers used for real-time PCR are shown in Table 6. For the urine study, standard plasmids were included in the real-time PCR to generate standard curves based on copy number (10^5^, 10^4^, 10^3^, 10^2^, and 10) and threshold cycle (Ct) values. To construct the standard plasmids, a 360 bp sequence (that included the PCR target region) derived from each target gene was synthesized and ligated into pUC57 plasmid DNA (GenScript, Piscataway, NJ). The synthesized target regions were confirmed by capillary sequencing. The Ct values for each gene were plotted on a standard curve to calculate the copy number. All samples were run in triplicate. The Quant-iT RiboGreen RNA Reagent Quant-iT RiboGreen RNA Kit (Invitrogen, Grand Island, NY) were used to measure the concentration of total cell-free RNAs purified from the urine samples.

**Table 6:** Sequence, and amplicon size of genes used in the study

Gene symbol	Primer	Amplicon Size (bp)
CDC20	S: CTG AAC CTT GTG GAT TGG AG	79
	AS: ATG TCA CCA GAG CTT GCA CT	
TOP2A	S: GAC TGT CTG TTG AAA GAA TC	87
	AS: ATT CCA CAG AAC CAA TGT AG	
ESM1	S: GCA AGA GGA CAG TGC TCG AC	95
	AS: GCC ATC CAT GCC TGA GAC TG	
UBE2C	S: CCA CAG TGA AGT TCC TCA CG	96
	AS: ACA GGG CAG ACC ACT TTT CC	
CA9	S: AGT GAA GAG GAT TCA CCC AGA	101
	AS: GGC TTA ACT TCA GGT AGA TCC	

TS, sense; AS, anti-sense

### Statistical analysis

The levels of urinary cell-free RNAs were compared between the BC and control groups using nonparametric methods because the expression data were not normally distributed and could not always be transformed to achieve normality. Receiver operating characteristic (ROC) curves were used to evaluate the diagnostic performance of the candidate urinary cell-free RNAs, and the optimal cut-off points for candidate marker selection were determined based on the highest combined sensitivity and specificityvalues obtained from ROC curve analysis. The diagnostic value of the urinary cell-free RNAs was determined using binary logistic regression models. Statistical analysis was performed using IBM SPSS Statistic ver. 21.0 (IBM Corp. Armonk, NY) and a *p*-value < 0.05 was considered significant.

## References

[1] Burger M, Catto JW, Dalbagni G, Grossman HB, Herr H, Karakeiwicz P, Kassouff W, Kiemeney LA, La Vecchia C, Shariat S, Lotan Y (2013). Epidemiology and risk factors of urothelial bladder cancer. Eur Urol.

[2] van der Aa MN, Steyerberg EW, Sen EF, Zwarthoff EC, Kirkels WJ, van der Kwast TH, Essink-Bot ML (2008). Patients' perceived burden of cystoscopic and urinary surveillance of bladder cancer: a randomized comparison. BJU Int.

[3] Lotan Y, Roehrborn CG (2003). Sensitivity and specificity of commonly available bladder tumor markers versus cytology: results of a comprehensive literature review and meta-analyses. Urology.

[4] Simon MA, Lokeshwar VB, Soloway MS (2003). Current bladder cancer tests: unnecessary or beneficial?. Crit Rev Oncol Hematol.

[5] Kim WT, Park K, Cho NH, Ham WS, Lee JS, Ju HJ, Kwon YU, Choi YD (2009). Comparison of the Efficacy of Urine Cytology, Nuclear Matrix Protein 22 (NMP22), and Fluorescence *in Situ* Hybridization (FISH) for the Diagnosis of Bladder Cancer. Korean J Urol.

[6] Kim SM, Kang HW, Kim WT, Kim YJ, Yun SJ, Lee SC, Kim WJ (2013). Cell-Free microRNA-214 From Urine as a Biomarker for Non-Muscle-Invasive Bladder Cancer. Korean J Urol.

[7] Jin X, Yun SJ, Jeong P, Kim IY, Kim WJ, Park S (2014). Diagnosis of bladder cancer and prediction of survival by urinary metabolomics. Oncotarget.

[8] Yun SJ, Jeong P, Kang HW, Kim YH, Kim EA, Yan C, Choi YK, Kim D, Kim JM, Kim SK, Kim SY, Kim ST, Kim WT (2015). Urinary MicroRNAs of Prostate Cancer: Virus-Encoded hsv1-miRH18 and hsv2-miR-H9-5p Could Be Valuable Diagnostic Markers. Int Neurourol. J.

[9] Gahan PB, Swaminathan R (2008). Circulating nucleic acids in plasma and serum. Recent developments. Ann NY Acad Sci.

[10] Casadio V, Calistri D, Tebaldi M, Bravaccini S, Gunelli R, Martorana G, Bertaccini A, Serra L, Scarpi E, Amadori D, Silvestrini r, Zoli W (2013). Urine cell-free DNA integrity as a marker for early bladder cancer diagnosis: preliminary data. Urol Oncol.

[11] Yun SJ, Yan C, Jeong P, Kang HW, Kim YH, Kim EA, Lee OJ, Kim WT, Moon SK, Kim IY, Choi YH, Kim WJ (2015). Comparison of mRNA, Protein, and Urinary Nucleic Acid Levels of S100A8 and S100A9 between Prostate Cancer and BPH. Ann Surg Oncol.

[12] Jahr S, Hentze H, Englisch S, Hardt D, Fackelmayer FO, Hesch RD, Knippers R (2001). DNA fragments in the blood plasma of cancer patients: quantitations and evidence for their origin from apoptotic and necrotic cells. Cancer Res.

[13] Pu XY, Wang ZP, Chen YR, Wang XH, Wu YL, Wang HP (2008). The value of combined use of survivin, cytokeratin 20 and mucin 7 mRNA for bladder cancer detection in voided urine. J Cancer Res Clin Oncol.

[14] Bryzqunova OE, Skvortsova TE, Kolesnikova EV, Starikov AV, Rykova EY, Vlassov VV, Laktionov PP (2006). Isolation and comparative study of cell-free nucleic acids from human urine. Ann NY Acad Sci.

[15] Okamoto Y, Ozaki T, Miyazaki K, Aoyama M, Miyazaki M, Nakagawara A (2003). UbcH10 is the cancer-related E2 ubiquitin-conjugating enzyme. Cancer Res.

[16] Chou CP, Huang NC, Jhuang SJ, Pan HB, Peng NJ, Cheng JT, Chen CF, Chen JJ, Chang TH (2014). Ubiquitin-conjugating enzyme UBE2C is highly expressed in breast microcalcification lesions. PLoS One.

[17] Pallante P, Berlingieri MT, Troncone G, Kruhoffer M, Orntoft TF, Viglietto G, Caleo A, Migliaccio I, Decaussin-Petrucci M, Santoro M, Palombini L, Fusco A (2005). UbcH10 overexpression may represent a marker of anaplastic thyroid carcinomas. Br J Cancer.

[18] Wagner KW, Sapinoso LM, El-Rifai W, Frierson HF, Butz N, Mestan J, Hofmann F, Deveraux QL, Hampton GM (2004). Overexpression, genomic amplification and therapeutic potential of inhibiting the UbcH10 ubiquitin conjugase in human carcinomas of diverse anatomic origin. Oncogene.

[19] Shen Z, Jiang X, Zeng C, Zheng S, Luo B, Zeng Y, Ding R, Jiang H, He Q, Guo J, Jie W (2013). High expression of ubiquitin-conjugating enzyme 2C (UBE2C) correlates with nasopharyngeal carcinoma progression. BMC Cancer.

[20] Matsumoto A, Ishibashi Y, Urashima M, Omura N, Nakada K, Nishikawa K, Shida A, Takada K, Kashiwagi H, Yanaga K (2014). High UBCH10 protein expression as a marker of poor prognosis in esophageal squamous cell carcinoma. Anticancer Res.

[21] Morikawa T, Kawai T, Abe H, Kume H, Homma Y, Fukayama M (2013). UBE2C is a marker of unfavorable prognosis in bladder cancer after radical cystectomy. Int J Clin Exp Pathol.

[22] Fristrup N, Birkenkamp-Demtroder K, Reinert T, Sanchez-Carbayo M, Segersten U, Malmstrom PU, Palou J, Alvarez-Mugica M, Pan CC, Ulhoi BP, Borre M, Orntoft TF, Dyrskjot L (2013). Multicenter validation of cyclin D1, MCM7, TRIM29, and UBE2C as prognostic protein markers in non-muscle-invasive bladder cancer. Am J Pathol.

[23] Payne SR, Serth J, Schostak M, Kamradt J, Strauss A, Thelen P, Model F, Day JK, Liebengerg V, Morotti A, Yamamura S, Lograsso J, Sledziewski A, Semionow A (2009). DNA methylation biomarkers of prostate cancer: confirmation of candidates and evidence urine is the most sensitive body fluid for non-invasive detection. Prostate.

[24] Goessl C, Muller M, Heicappell R, Krause H, Miller K (2001). DNA-based detection of prostate cancer in blood, urine, and ejaculates. Ann NY Acad Sci.

[25] Tsui NB, Ng EK, Lo YM (2002). Stability of endogenous and added RNA in blood specimens, serum, and plasma. Clin Chem.

[26] Hanke M, Kausch I, Dahmen G, Jocham D, Wamecke JM (2007). Detailed technical analysis of urine RNA-based tumor diagnostics reveals ETS2/urokinase plasminogen activator to be a novel marker for bladder cancer. Clin Chem.

